# Porcelain Aorta in a Young Person Living with HIV Who Presented with Angina

**DOI:** 10.3390/diagnostics12123147

**Published:** 2022-12-13

**Authors:** Mircea Bajdechi, Alexandru Scafa-Udriste, Vlad Ploscaru, Lucian Calmac, Teodor Bajeu, Adriana Gurghean, Sorin Rugina

**Affiliations:** 1 Doctoral School of Medicine, “Ovidius” University of Constanta, 900470 Constanta, Romania; 2 Faculty of Medicine, University of Medicine and Pharmacy “Carol Davila” of Bucharest, 050474 Bucuresti, Romania; 3 Emergency Clinical Hospital of Bucharest, 014461 Bucuresti, Romania; 4 Doctoral School, Politehnica University of Bucharest-Applied Chemistry and Materials Science, 060042 Bucuresti, Romania

**Keywords:** people living with HIV, porcelain aorta, coronary syndrome, angina, antiretroviral therapy

## Abstract

People living with human immunodeficiency virus have an increased cardiovascular risk due to higher prevalence of traditional risk factors, such as smoking, dyslipidemia, hypertension, diabetes, or obesity, and particular risk factors, such as inflammation, endothelial dysfunction, and antiretroviral therapy. Thus, people living with human immunodeficiency virus can develop accelerated atherosclerosis. The incidence of coronary artery disease in these patients may be twice as high compared with that of HIV-negative individuals with similar characteristics. “Porcelain aorta” is a term used to describe extensive circumferential calcification of the thoracic aorta. The pathophysiology of porcelain aorta is not fully understood. We present a case of a young man who was a smoker and living with HIV since childhood, without other traditional cardiovascular risk factors, who presented to the emergency room with a positive stress test for myocardial ischemia. Transthoracic echocardiography revealed normal regional and global myocardial wall motion, ascending aorta ectasia, and moderate aortic regurgitation. Coronary angiography showed a critical calcified proximal left anterior descending artery stenosis and an important calcification of the thoracic aorta. Therefore, the most important challenge was the management of coronary syndrome in a young person living with HIV, with associated porcelain aorta and aortic regurgitation.

## 1. Introduction

People living with human immunodeficiency virus (PLWHIV) live longer now due to highly active antiretroviral therapy (HAART), but they have an increased cardiovascular risk due to higher incidence of traditional risk factors, such as smoking, dyslipidemia, hypertension, diabetes, or obesity, and particular risk factors, such as inflammation, endothelial dysfunction, coagulation abnormalities, selenium deficiency, and, last but not least, antiretroviral therapy [1,2,3]. 

It is anticipated that by the year 2030, most of the PLWHIV will be over the age of 50 years old and 78% of them will be suffering from cardiovascular disease [4]. Coronary artery disease (CAD) became the most important cardiac disease in people living with HIV in developed countries [1]. 

“Porcelain aorta” (PA) is a term used to describe extensive circumferential calcification of the thoracic aorta, such that it precludes safe cross-clamping or entry to the ascending aorta [5]. The incidence of porcelain aorta is higher in patients with aortic stenosis, coronary artery disease, or in older patients. The prevalence of PA has been reported as 0.6 to 7.5% in patients requiring cardiac surgery [6,7,8,9] and 7.5% in patients with aortic stenosis [10]. There are no studies on young patients with porcelain aorta. 

A relationship between HIV infection and porcelain aorta has not been found. There are studies have shown a strong association between other systemic inflammatory diseases (Takayasu arteritis, systemic lupus erythematosus, and rheumatoid arthritis) and porcelain aorta [5,11,12]. The atherosclerosis as a result of an inflammatory response involving the tunica intima can occur through one of two mechanisms. The second mechanism is a non-atherosclerotic mechanism, which involves calcification of mainly the medial layer of the aorta, in the absence of atheroma [5,11]. Aortic atheromatosis is often associated with coronary artery disease. An observational study that performed 175 consecutive CT aortograms reported an absence of aortic calcification in 33 patients (18.9%), coronary artery disease in 90 patients (51.4%), and porcelain aorta in 1 patient (0.6%) [9].

The management of patients with porcelain aorta and aortic regurgitation who present with acute or chronic coronary syndrome caused by severe calcified lesion of the proximal left anterior descending (LAD) artery can be difficult. 

## 2. Case Report

**Figure 1:**
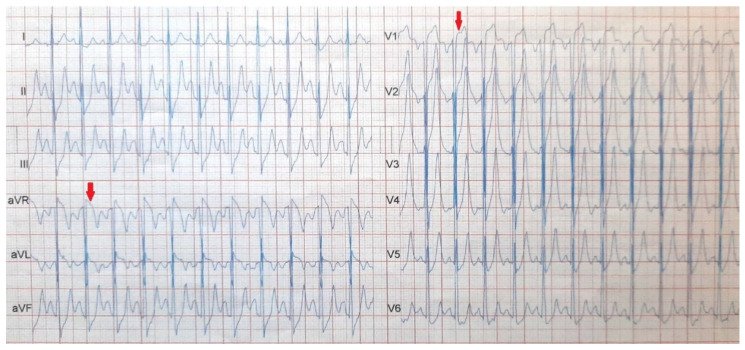
Bruce stress test: at 8 min and 20 s. During the 3rd stage, the test was stopped due to angina and the electrocardiogram (ECG) findings at 150 bpm showed diffuse ST-segment depression and ST-segment elevation in aVR and V1 > 3 mm.

A 33-year-old Caucasian man presented with stable angina and a positive Bruce stress test for ischemia (Figure 1). Except for smoking, the patient did not have other common cardiovascular risk factors. He was treated with acetyl salicylic acid, atorvastatin, metoprolol succinate, and isosorbide mononitrate for coronary artery disease for two months. 

The patient was infected with HIV-1 by iatrogenic procedures and he is part of “The Romanian HIV cohort”, which includes over 5000 patients that were infected during childhood between 1987 and 1989. At the moment, he is on antiretroviral therapy (ART) that includes two nucleoside reverse-transcriptase inhibitors (NRTIs), tenofovir and abacavir, and one integrase inhibitor (II), raltegravir. At the time of this study, the patient had an undetectable viral load, the cluster of differentiation number (CD4) was 1462 cells/mm^3^, and the CD4/CD8 ratio was 1.44.

**Figure 2:**
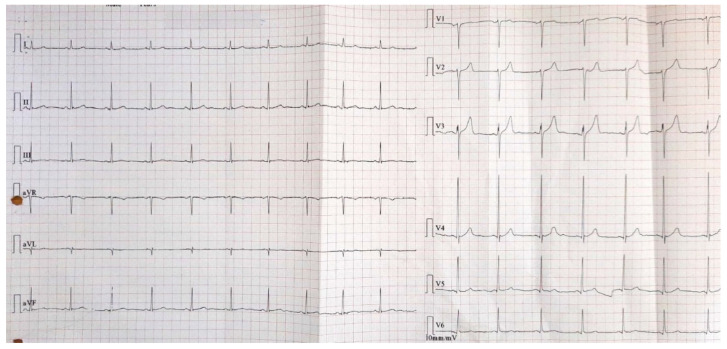
Resting standard ECG: sinus rhythm, normal QRS axis, ventricular rate 70 bpm, Q wave in aVL, and without any ST-segment or T-wave abnormalities.

His physical examination and laboratory tests were unremarkable (no elevated myocardial necrosis markers, normal cholesterol values, estimated glomerular filtration rate of 118.1 mL/min/1.73^2^). The resting standard electrocardiogram showed sinus rhythm, normal QRS axis, ventricular rate of 70 bpm, and Q wave in aVL, without any ST segment or T-wave abnormalities (Figure 2).

**Figure 3:**
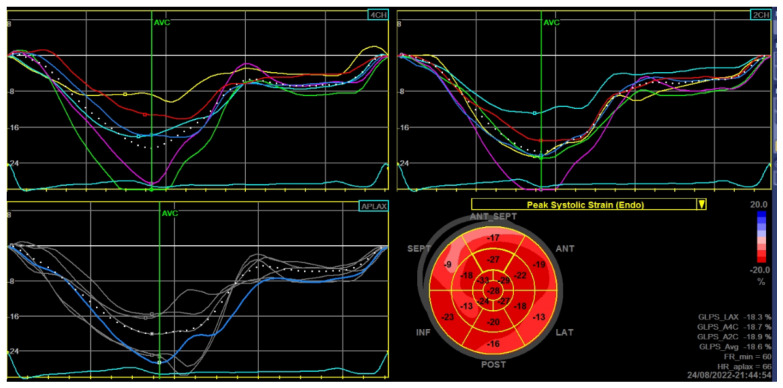
Global longitudinal strain: mildly reduced peak systolic strain (Endo) - global longitudinal peak strain average (GLPS_Avg) of −18.6%.

**Figure 4:**
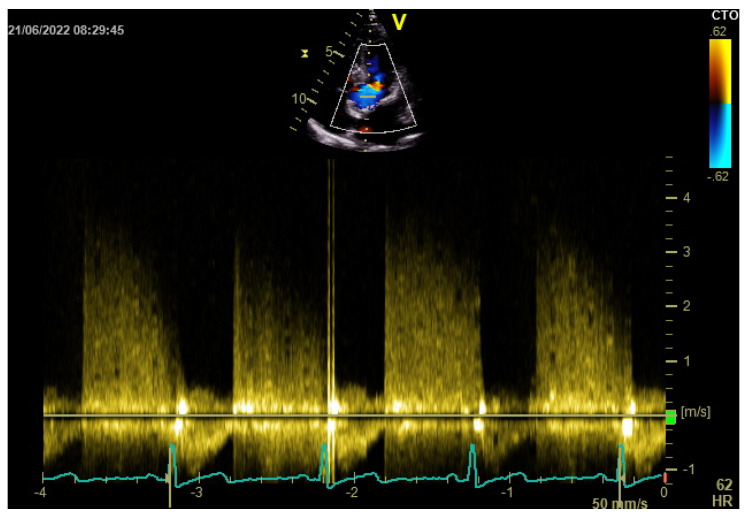
Transthoracic echocardiography: color Doppler echocardiographic images in apical five chamber view and continuous wave Doppler spectrum from the left ventricular outflow tract showed aortic regurgitation flow. The pressure halftime (PHT) was 0.412 seconds.

Transthoracic echocardiography showed a left ventricular cavity of normal size with normal systolic function (left ventricular ejection fraction of 55% without wall motion abnormalities, but mildly reduced global longitudinal strain (GLS −18.6%) (Figure 3), ascending aorta ectasia, and moderate aortic regurgitation (Figure 4).

**Figure 5:**
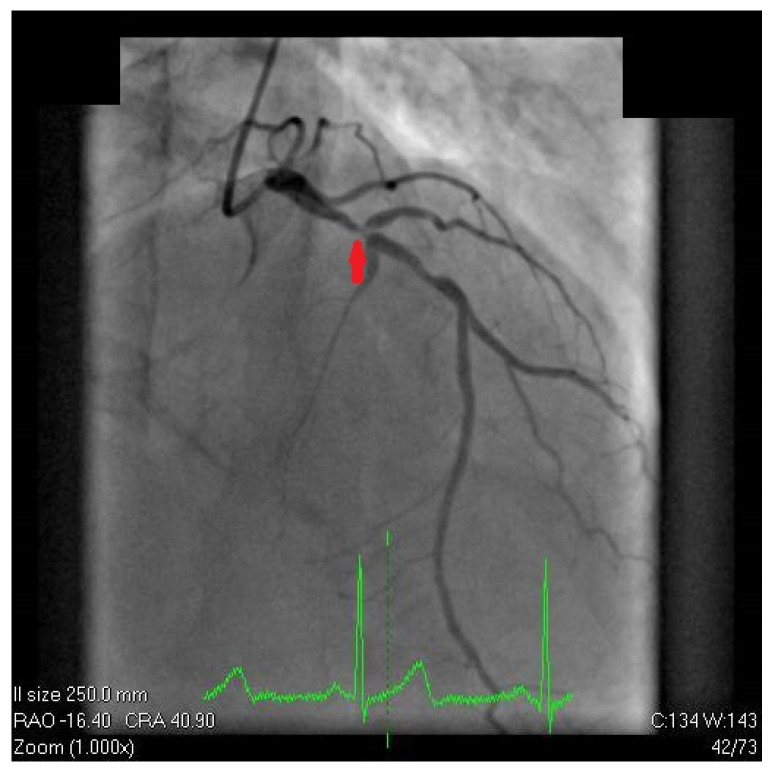
Coronary angiography. Cranial projection of the left coronary artery revealed significant calcified lesion in the proximal left anterior descending artery involving the principal diagonal branch (Medina 1,1,1 with TIMI3 flow).

**Figure 6:**
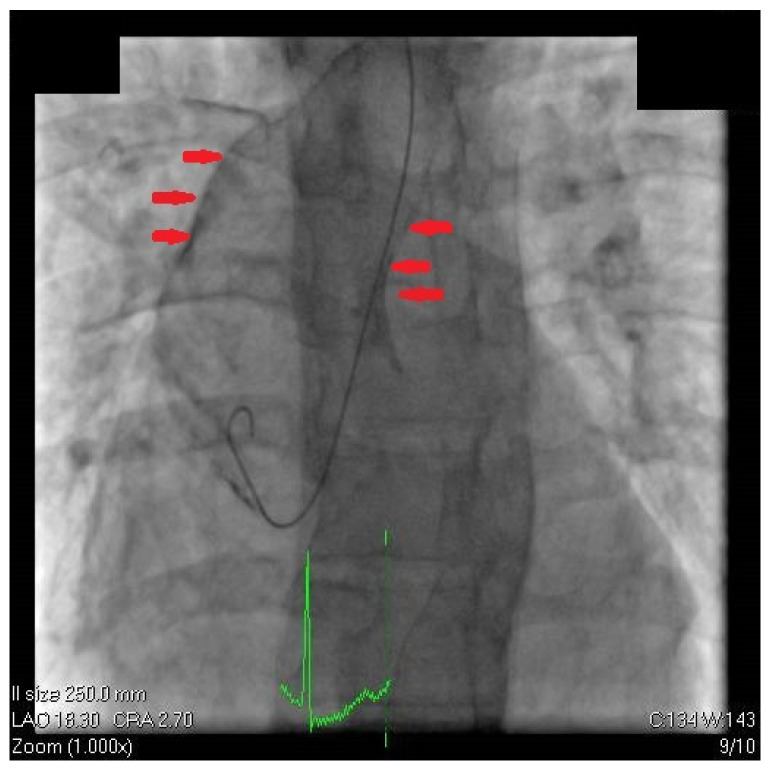
Fluoroscopy during coronary angiography detected aortic calcification highly suggestive of porcelain aorta (PA). The red arrows point the aortic walls.

The patient was admitted to the cardiology department due to a high-risk cardiovascular profile, positive Bruce stress test, and echocardiographic abnormalities. Afterward, the patient qualified for coronary angiography by transradial access, which revealed a significant calcified lesion in the proximal left anterior descending artery involving the principal diagonal branch (Medina 1,1,1 with TIMI 3 flow) (Figure 5). The fluoroscopy during coronary angiography detected severe aortic calcification compatible with porcelain aorta (PA) (Figure 6). As the patient did not have increased myocardial necrosis markers and did not require immediate angioplasty, we decided that he should be admitted to the coronary intensive care unit, in order to subsequently carry out additional investigations and to decide on revascularization options.

**Figure 7:**
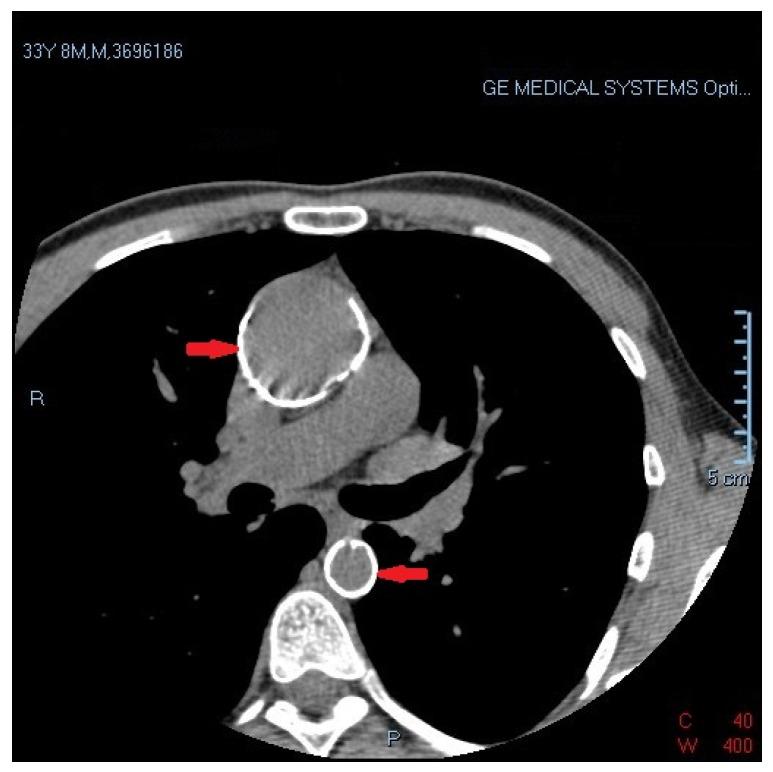
Computed tomography (CT) confirmed severe ascending and descending aortic calcification compatible with porcelain aorta.

The decision between the revascularization options was based on the SYNTAX Score II, which showed significantly higher 4-year mortality for percutaneous coronary intervention (PCI) (1.6%) vs. coronary artery bypass graft (CABG) (0.6%). To predict in-hospital mortality after cardiac surgery, we calculated the EuroSCORE II, which showed a 2% risk of in-hospital mortality. Beyond these risk scores, this patient had certain particularities such as: age, HIV infection and porcelain aorta (Figure 7).

**Figure 8:**
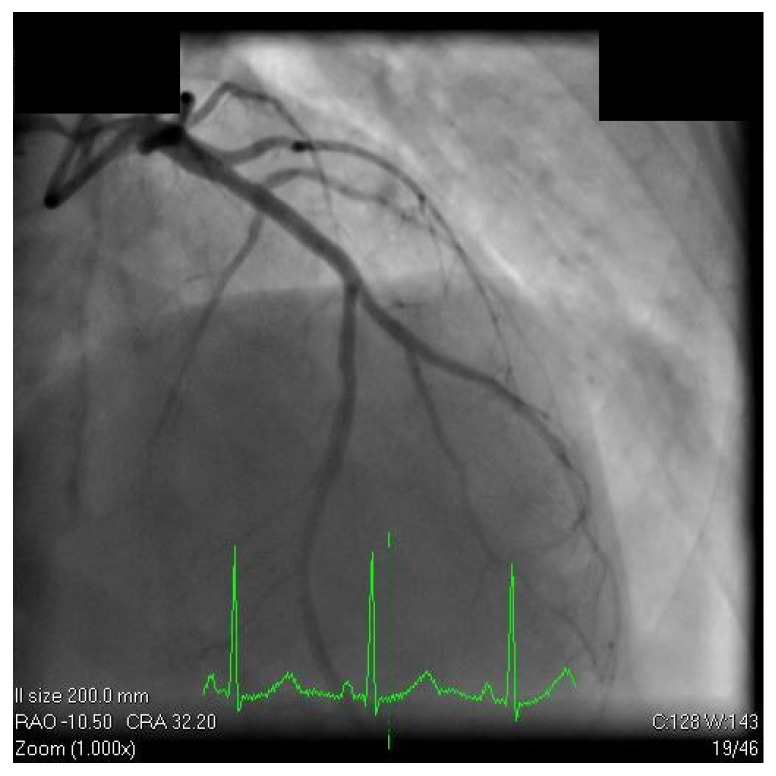
Coronary angiography postangioplasty. Cranial projection of the left coronary artery showed improved blood flow to the LAD and to the diagonal branch, without any intraprocedural complications.

According to current guidelines, the clinical decision was made by a “heart team” consisting of an interventional cardiologist, a cardiac surgeon, and a non-interventional cardiologist. After sharing the decision with the patient and obtaining informed consent, he was qualified for percutaneous coronary intervention. The patient underwent angioplasty by radial access using a 6F catheter, an angioplasty guidewire (Nitinol Abbott Hi-Torque Balance Middleweight Guidewire) and another guidewire in the principal diagonal branch. First, we predilated the LAD artery lesion with a semi-compliant balloon (Mini Trek Abbott 2.5 × 15 mm, 16 atmosphere) and the proximal segment of diagonal branch with a semi-compliant balloon (Mini Trek Abbott 2 × 20 mm, 20 atmosphere), then we implanted an everolimus-eluting stent (Xience 3 × 33 mm 16 atmosphere) to the proximal LAD segment, culminating in the proximal optimization technique with a non-compliant balloon (Emerge Boston Scientific 3 × 15 mm, 20 atmosphere). The angiographic result was satisfactory, with improvement in the blood flow to the LAD and to the diagonal branch, without any intraprocedural complications (Figure 8).

The patient was monitored for one more day and was discharged with the following medication: acetylsalicylic acid 75 mg once daily, ticagrelor 90 mg twice daily (for 12 months minimum), metoprolol succinate 150 mg per day, atorvastatin 80 mg once daily and isosorbide mononitrate 40 mg once daily, along with the recommendation to quit smoking. Drug–drug interactions between this treatment and antiretroviral therapy were not found. However, the infectious disease specialist decided to switch the treatment to a drug containing three active substances, bictegravir, emtricitabine, and tenofovir alafenamide. The removal of abacavir was decided due to its proinflammatory and prothrombotic effect, which could have led to accelerated atherosclerosis. At his 6-month follow-up, the patient had good clinical status, without angina, with a good effort tolerance. The electrocardiographic findings and echocardiographic parameters did not show significant changes. During these months, the newly introduced antiretroviral regimen controlled the infection well, the viremia remained undetectable, the cluster of differentiation number (CD4) was 1277 cells/mm^3^, and the CD4/CD8 ratio was 1.48.

## 3. Discussion

It is anticipated that by the year 2030, 73% of people living with HIV will be over the age of 50 and 78% of them will suffer from cardiovascular disease [4].

Cardiovascular disease is the leading cause of death in people living with HIV, accounting for around 11% of the total deaths [13] and is reported in proportions of 6.5% in Europe [14], 8% in France [15], and 15% in North America [1].

The etiology of coronary artery disease in PLWHIV is multifactorial and includes the increased prevalence of traditional risk factors, use of illicit drugs, as well as particular risk factors, with chronic inflammation and activation of the immune system being key factors leading to accelerated and diffuse atherosclerosis [3]. Exposure of combination antiretroviral therapy plays a role in the exacerbation of cardiovascular risk factors [16,17]. Certain antiretrovirals, such as those that form the protease inhibitors class, widely used in the past, can lead to an increased cardiovascular risk through significant disorders of lipid and carbohydrate metabolism. Exposure to some nucleoside reverse-transcriptase inhibitors (NRTIs), such as abacavir, can be associated with increased risk of myocardial infarction [2,18,19,20]. Experimental studies demonstrated that, in mice, abacavir had proinflammatory and prothrombotic effects by increasing the interactions between thrombocytes, leucocytes, and endothelium, favoring platelet aggregation and thus increasing the risk of acute myocardial infarction [21]. Other studies presented results that do not confirm an association between abacavir and myocardial infarction [22,23]. Although this patient was on a protease inhibitor regimen for over 15 years, no high cholesterol values were found in the last 12 years (115 mg/dL in 2010 and 116 mg/dL in 2022). In the current literature, the direct implications of abacavir in increasing the risk of myocardial infarction are uncertain; therefore, we did not find studies that recommend stopping abacavir in patients with coronary artery diseases. The European AIDS Clinical Society guidelines highlight that abacavir can potentiate platelet activation, potentially increasing cardiovascular risk, and recommend caution in its use in patients with a cardiovascular risk of over 10% [24]. In this particular case (the association of a coronary artery disease with another severe atherosclerotic determination, porcelain aorta), we decided that it would be beneficial for the patient to replace abacavir with another nucleoside reverse transcriptase inhibitor (NRTI), emtricitabine, which has equivalent therapeutic benefits without proatherogenic effects. The three revascularization options discussed by the heart team were: percutaneous coronary intervention with a drug-eluting stent (with the risk of stent underexpansion), coronary artery bypass grafting (with an increased risk of complications secondary to the porcelain aorta), or beating heart coronary artery bypass grafting. After sharing the decision with the patient, we decided to perform percutaneous coronary intervention with a drug-eluting stent. Recent data have shown that in-hospital risk of cardiac and cerebrovascular events in patients with acute coronary syndrome and HIV infection is similar to that of the general population, but the risk of recurrence of ischemic events and the readmission for decompensation of heart failure after a first episode of coronary syndrome is higher among HIV patients [25]. Therefore, patients with HIV have an increased risk of developing coronary artery disease and ischemic recurrences after acute coronary syndrome [1,26,27] and can have severe complications, such as intrastent restenosis or early coronary artery bypass revascularization failure [28], compared with HIV-negative patients.

Porcelain aorta (PA) is a rare entity in the general population, its prevalence reaching 1.2% in patients who underwent coronary artery bypass grafting [6], and is associated with several disease processes, including atherosclerosis; chronic systemic inflammatory diseases such as Takayasu arteritis, systemic lupus erythematous, and rheumatoid arthritis; chronic kidney disease; or mediastinal radiotherapy [11]. There are no studies reporting its incidence in young patients. The pathophysiology of PA is not fully understood. Two independent processes lead to the formation of aortic calcification: an atherosclerotic mechanism that occurs as a result of inflammatory response involving the tunica intima of the aortic wall due to atherosclerotic plaque development and a non-atherosclerotic mechanism that involves calcification of mainly the medial layer of the aorta in the absence of atheroma [5,11]. Regarding the possible link among HIV infection, antiretrovirals, and porcelain aorta, we found no data in the literature. HIV-related atherosclerosis is focused on macrophages and their decisive role in the inflammatory process and in plaque development [29]. An atherogenic molecular mechanism may be related to the interaction between HIV with host immune cells and endothelial cells, which leads to increased oxidative stress and the development of inflammatory cells and the inhibition of autophagy [29,30]. The patient was diagnosed more than ten years after being infected, when he had an advanced disease stage (CD4 165 cells/mm^3^ and viremia 16,300 copies/mL), which could have played a part in early atherosclerosis development. A prospective study of 361 stable angina pectoris patients who underwent chest spiral computed tomography showed that aortic calcification was present in 253 patients (70% of patients), and significant correlation was found between these patients and coronary calcification as expressed by coronary calcium score [31]. This patient has several particularities: young age, HIV-1 infection acquired by iatrogenic procedures during his childhood, porcelain aorta, and aortic ectasia, which led to moderate aortic regurgitation and coronary syndrome caused by severe calcified lesion of the proximal left anterior descending artery. These characteristics were very challenging for the management of this patient. Percutaneous coronary intervention with a drug-eluting stent was likely the best option to treat this case. This seems to be, to the best of our knowledge, the first case of porcelain aorta described in a young person living with HIV.

## 4. Conclusions

People living with HIV can develop accelerated and diffuse atherosclerosis, which can lead to severe vascular damage. Coronary artery disease has become an important cause of mortality in these patients. Non-invasive tests to detect myocardial ischemia should be studied in order to detect early stage disease. A correlation between porcelain aorta and HIV infection has not been reported in the literature.

## Data Availability

Not applicable.
